# Evaluation of Self-Care Activities and Quality of Life in Patients With Type 2 Diabetes Mellitus Treated With Metformin Using the 2D Matrix Code of Outer Drug Packages as Patient Identifier: Protocol for the DePRO Proof-of-Concept Observational Study

**DOI:** 10.2196/21727

**Published:** 2021-01-11

**Authors:** Christian Mueller, Isabel Schauerte, Stephan Martin

**Affiliations:** 1 Pharmaceuticals Medicine, Pharmaceuticals Medical Excellence & Innovation Management, Data Generation Bayer Vital GmbH Leverkusen Germany; 2 Institut Dr Schauerte Munich Germany; 3 Westdeutsches Diabetes- und Gesundheitszentrum Düsseldorf Germany

**Keywords:** self-care activities, quality of life, type 2 diabetes mellitus, patient-reported outcome measures, digital observational study, bring your own device

## Abstract

**Background:**

Diabetes mellitus (DM) is one of the most common noncommunicable diseases. DM has a substantial negative impact on patients’ quality of life, which is measured using a variety of diabetes-specific measures covering multiple aspects of patients’ psychological state, behavior, and treatment satisfaction. A fully digital data collection system, including patient identification, would represent a substantial advance in how these patient-reported outcome (PRO) data are measured. Within the European Union, one way to identify patients without the involvement of health care professionals is to use the unique 2D matrix codes on the packaging of prescription medication—for example, metformin, the recommended initial treatment for patients with type 2 DM (T2DM).

**Objective:**

In the DePRO study we aim to (1) describe the self-care activities of patients with T2DM using metformin-containing medication; (2) describe the self-reported health status (eg, presence of diabetes complications and quality of life) of these patients; (3) describe associations between self-care activities and demographics and disease characteristics; and (4) assess the usability of the my ePRO app.

**Methods:**

DePRO is an observational, multicenter, cross-sectional, digital, patient-driven study conducted in Germany. Patients with a prescription for a metformin-containing medication will be given a postcard by their pharmacist, which will include a download link for the my ePRO app. In total, 12 diabetes-focused pharmacies, selected to represent urban and rural areas, will be recruited. Participants will use their own mobile device (bring your own device) to download the my ePRO app and access the DePRO study, for which they can register using the 2D matrix code on their medication. An electronic informed consent form will be displayed to the patients and only after giving consent will patients be able to complete the study questionnaires. The PRO instruments used in the study are the Summary of Diabetes Self-Care Activities Scale, the Diabetes Treatment Satisfaction Questionnaire, and the 5 level, 5-dimension EuroQol Questionnaire. Patients will also be asked to complete a questionnaire with items addressing demographics, patient characteristics, disease history, complications, and concomitant medications. Data will be transferred to the study database by the app upon completion of each questionnaire. Statistical analyses of primary and secondary endpoints will be exploratory and descriptive.

**Results:**

Enrollment began in June 2020. The estimated study completion date is December 31, 2020, and the planned sample size is 300 patients.

**Conclusions:**

The DePRO study uses completely digital data collection, including authentication of eligible patients and completion of the study questionnaires. Therefore, the design of the DePRO study represents a substantial advance in the evaluation of the digital capturing of PRO data.

**Trial Registration:**

ClinicalTrials.gov NCT04383041; https://clinicaltrials.gov/ct2/show/NCT04383041

**International Registered Report Identifier (IRRID):**

PRR1-10.2196/21727

## Introduction

### Background

Diabetes mellitus (DM) is one of the most common noncommunicable diseases, with a growing global prevalence which is impacting negatively on the sustainability of health care systems [1]. According to the International Diabetes Federation, DM affected 463 million people globally in 2019, a number forecast to grow to 578 million by 2030 [2]. An estimated 8.5% of the adult population in Europe has DM, with national prevalence rates ranging from 2.4% to almost 15% [3]. DM has a high negative impact on society as a result of the severe comorbidities and complications associated with the disease [4]. Several studies have estimated that 90%-95% of all patients with DM are affected by type 2 DM (T2DM) and that the prevalence of T2DM will continue to increase due to population aging [2,5]. The complications of DM affect patients’ quality of life [6,7] and increase the risk of negative events such as emergency department visits, hospitalization, and death, with consequences for health care costs and the sustainability of health care systems [8]. Clinical recommendations refer to diabetes self-care and self-management as key to preventing disease complications and maintaining patients’ health and quality of life over time [9,10].

Assessment of psychosocial functioning and health-related quality of life has gained prominence in the care and treatment of patients with diabetes over the past decade. This has resulted in the development of a variety of diabetes-specific measures covering multiple aspects of patients’ psychological state, behavior, and treatment satisfaction. These include, for example, the Summary of Diabetes Self-Care Activities Scale (SDSCA) and the Diabetes Treatment Satisfaction Questionnaire (DTSQ) [11,12].

It has been estimated that there were 6.9 million patients with T2DM in Germany in 2015 [13]. The recommended initial treatment for patients with T2DM is metformin [14]; in 2018, a total of 606 million daily doses of metformin were prescribed in Germany, corresponding to 160 different medications provided by 25 marketing authorization holders [15].

Within the European Union the Falsified Medicine Directive (Directive 2011/62/EU) defines “the characteristics and technical specifications of the unique identifier of the safety features … that enables the authenticity of medicinal products to be verified and individual packs to be identified” [16]. This 2D matrix code on the outer packaging of medication contains a country code, a product code, a serial number, the expiry date, and the charge of the medication. Once a patient has possession of the package, the 2D matrix code is no longer an identifier only of the medication, but also of the patient as the user of this medication.

### Objectives

In the DePRO study we aim to (1) describe the self-care activities of patients with T2DM using metformin; (2) describe the self-reported health status (eg, presence of diabetes complications and quality of life) of these patients; (3) describe associations between self-care activities and demographics and disease characteristics; and (4) assess the usability of the my ePRO app.

## Methods

### Study Design

**Figure 1:**
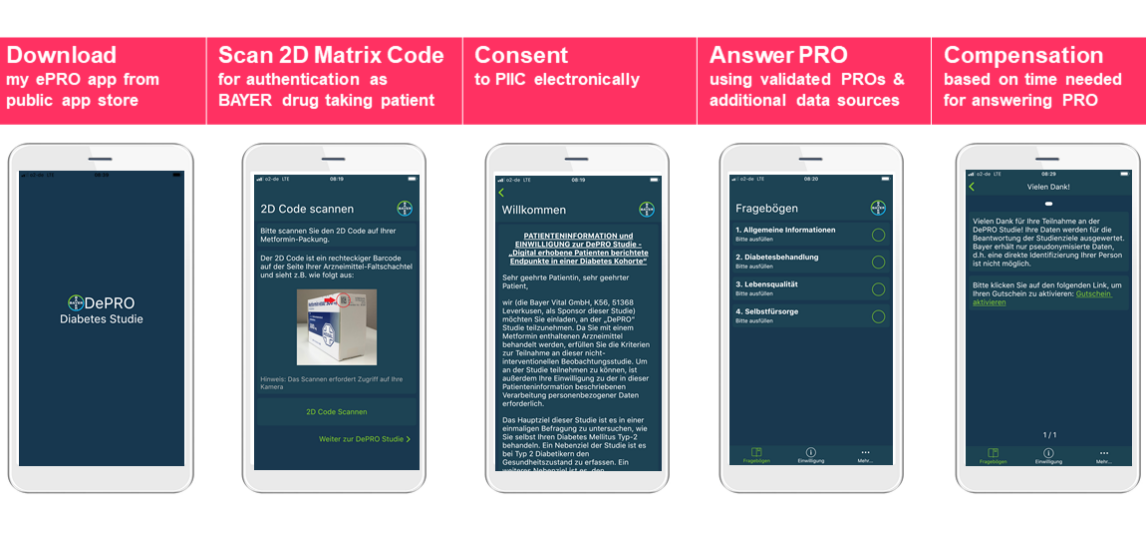
Recruitment and data capturing process with the my ePRO app. EQ-5D-5L: 5-level, 5-dimension EuroQol Questionnaire; PIIC: patient information and informed consent form; PRO: patient-reported outcome.

DePRO is an observational, multicenter, cross-sectional, digital, patient-driven study conducted in Germany. Patients with a prescription for a metformin-containing medication will be eligible for participation and will be consecutively invited to participate. Patients receiving metformin-containing medication will be given a postcard by their pharmacist, which will include a download link for the my ePRO app. Patients will conduct the entire study without any support from the pharmacist. Participants will use their own mobile device (smartphone or tablet) to download the my ePRO app and will be directed through the app to the DePRO study. For registration and authentication they will use the unique 2D matrix code on their metformin-containing drug package. An electronic informed consent form will be displayed to the patients and only after giving consent will patients be able to complete the study questionnaires. After completing the questionnaires and uploading a picture of the metformin-containing drug package, patients will receive compensation for their time, in the form of a voucher or a donation to charity (Figure 1 and Multimedia Appendix 1). Data will be transferred to the study database by the app upon completion of each questionnaire. The final data analysis for the study will be performed by a contract research organization, Institut Dr. Schauerte, Munich, Germany (IDS).

### Ethical Considerations

The study protocol has been approved by the Ethics Committee of the Medical Association North Rhine (approval no. 2020084).

### Patients

Adult patients with a valid prescription for a metformin-containing medication will be enrolled in a public pharmacy after the decision for treatment with metformin has been made by their treating physician and informed consent is given by patients. No restrictions on potential eligibility for the DePRO study will be applied.

### Study Device: my ePRO App

The my ePRO app is a data capture tool which can be used for either stand-alone studies or piggy-backed on randomized controlled trials and observational studies to track patients’ health status. The app uses standardized, validated patient-reported outcome (PRO) instruments. Furthermore, the app has the capacity to gather additional data (physical activity, face and voice recognition, weather data) based on the research questions in each study. Users are provided with a unique app account, free of charge. By using the quick response (QR) code scanner built into the app patients can scan either a study-specific QR code provided on a patient information leaflet or the medication-specific 2D matrix code on the outer packaging of their medication. The latter option leads to company-specific pages where studies and the respective consent forms are offered. The my ePRO app is available for the most common device types (smartphones and tablets) running the most widespread mobile operating systems (Android and iOS). The my ePRO app was co-developed by IDS and Bayer, and is hosted by IDS.

### Data Transfer and Processing

Generally, the app will be used online. When not connected to the internet (offline mode), the app stores the entered data so that no data loss occurs. Data are transferred to the database as soon as the device is online again.

### Data Collection and Outcome Measures

The treating physician will have prescribed a metformin-containing medication, but will not contribute to data collection during the study. The patient’s data and answers to the questionnaires will be captured directly by the patient in the relevant sections of the my ePRO app using their own mobile devices.

By scanning the 2D matrix code with the my ePRO app, the patient is linked to a unique central patient identification code, which is only used for study purposes. For the duration of the study and afterward, only the authorized contract research organization personnel at IDS are able to link the patient identification code to the medication package used. For collection of data on concomitant medication use, the app can be used to scan the barcodes on the outer packaging of both prescription and nonprescription drugs. The number of invited, participating and nonparticipating patients—as stated to the inviting pharmacist—and the reasons given for nonacceptance of study participation will be collected by the pharmacist in a recruitment log.

The PRO instruments used in the study are the SDSCA, the DTSQ, and the 5-level, 5‑dimension EuroQol Questionnaire (EQ-5D-5L). The SDSCA is a questionnaire which assesses levels of self-care in adults with diabetes and was developed by Toobert et al [11]. The DTSQ is used to assess patients’ satisfaction with their diabetes treatment [12]. The EQ-5D-5L is a widely used instrument for measuring generic health status using a 5-dimension descriptive system and a visual analog scale [17], both of which are included in the DePRO study.

To complete the clinical picture of each patient the following variables will be collected in a questionnaire (not previously validated) requesting patients’ self-reported age; sex; weight; height; geographic region (first 2 digits of postal code); concomitant medication; education level; family income; time from the diagnosis of T2DM; occurrence of diabetes microvascular complications (diabetic kidney disease, diabetic retinopathy, diabetic neuropathy, and diabetic foot); occurrence of diabetes macrovascular complications (coronary arterial disease and stroke); and glycated hemoglobin (Hb_A1c_; Table 1 and Multimedia Appendices 2 and 3).

**Table 1:** Data captured within DePRO study by origin.

Data captured		Data source	
		Patient	Pharmacist
**Study information**			
	Date and time of data collection within my ePRO app	X	
	Primary reason for discontinuation (if applicable; eg, consent withdrawn by patient)	X	
	Reasons for nonacceptance of study participation		X
	Picture of the metformin-containing drug package	X	
**Patient characteristics**			
	Demography (eg, age, sex, geographic region, education level, family income)	X	
	Patient characteristics (eg, height, weight)	X	
	Disease history	X	
	Microvascular complications (diabetic kidney disease, diabetic retinopathy, diabetic neuropathy, and diabetic foot)	X	
	Macrovascular complications (coronary arterial disease, stroke)	X	
	Glycated hemoglobin (Hb_A1c_)	X	
	Concomitant medication (QR^a^ code scan)	X	
**PRO^b^ results**			
	DTSQ^c^	X	
	SDSCA^d^	X	
	EQ-5D-5L^e^	X	
	EQ-5D VAS^f^	X	

^a^QR: quick response.

^b^PRO: patient-reported outcome.

^c^DTSQ: Diabetes Treatment Satisfaction Questionnaire.

^d^SDSCA: Summary of Diabetes Self-Care Activities.

^e^EQ-5D-5L: 5-level, 5-dimension EuroQol Questionnaire.

^f^VAS: visual analog scale.

### Statistical Analyses

#### Primary and Secondary Endpoints

Statistical analyses of primary and secondary endpoints will be exploratory and descriptive. The study is not designed to confirm or reject predefined hypotheses.

#### Patient Population Size

The DePRO study aims to assess the current self-care activities of patients with T2DM in Germany within a cross-sectional design by testing the feasibility of data collection with the my ePRO app. We will therefore recruit 12 diabetes-focused pharmacies, selected to represent urban and rural areas across Germany, to participate in the DePRO study. Based on a previously conducted feasibility assessment with these pharmacies, the mean number of patients filling a metformin-containing prescription per quarter is approximately 4500. Assuming a variability of ±15% per quarter, 5175 postcards providing the download code for the my ePRO app will be provided to the pharmacies, representing the maximum sample size. Therefore, a planned recruitment time of 3 months, which covers the typical treatment situation of patients with T2DM (at least quarterly visits are necessary to receive a metformin prescription), determines the sample size of the DePRO study. For sample size considerations, we assume that 7%-10% of invited patients will complete the study. We further assume that the SD of SDSCA would be 13 points, according to the SD observed in the study by Ausili et al [18]. Given this assumption, a sample size of 300 patients is required to obtain a 95% confidence interval of the mean level of self-care with a precision (ie, width of the interval) equal to 3 points.

## Results

The DePRO study uses completely digital data collection. All data are patient reported and are not verified by any health care professional. The recruitment of patients is not based on the diagnosis of an investigator, but on the consequence of a diagnosis—namely the prescription of an approved drug. By providing software (my ePRO app) to patients and authenticating with available hardware (the outer packaging of the medication, containing the 2D matrix code), it will be possible to collect data directly from patients.

Enrollment began in June 2020. The estimated study completion date is December 31, 2020.

## Discussion

### Rationale for Study Design

The DePRO study will not only evaluate PROs measured using validated instruments, but also describe the feasibility of a fully digital data capture workflow. The study focuses on data which can be easily and reproducibly generated by patients and do not need any further validation by health care professionals. It is not necessary for health care professionals to be part of the data collection workflow for PRO assessment. This has already been achieved in randomized controlled trials and observational studies, by offering ePRO tools which enable patients to complete PROs whenever and wherever they want. The innovation in the approach taken in the DePRO study is to also bypass the need for the involvement of health care professionals in the authentication of eligible patients. By using the 2D matrix code on the outer packaging of medication a valid and uniform authentication is possible across the European Union. This was not possible previously, because linear bar codes (the former standard for pharmaceuticals in the European Union) cannot encode dynamic data such as batch numbers and expiry dates and therefore do not provide a unique identifier for each package [19]. According to the Commission Delegated Regulation (European Union) 2016/161 [20], all prescription drugs (with specific exceptions such as radionuclide generators and precursors) are now required to bear a 2D matrix code on their packaging, allowing remote patient authentication across a range of indications. The methodology can be applied in longitudinal as well as cross-sectional studies (patients can scan multiple new packs of medication over time, according to the defined logic in the back end of the app and the objectives of each study). Furthermore, the sample size of future studies using this methodology would no longer be limited by the number of health care professionals willing to recruit patients, but by the number of prescriptions issued. There are analogies between the scalability of studies using the my ePRO app and the Apple Heart Study [21]. In both studies, a large quantity of hardware (Apple Watches/prescribed drugs) is available in the market and can be used to authenticate patients. The sponsor of a study provides publicly available software (Apple Heart Study app/my ePRO app) to patients. Patients enter data without the help of health care professionals. In the case of the Apple Heart Study, and potentially of the DePRO study, the participation rate is low (in the Apple Heart Study 419,297 patients were recruited from among more than 30 million device users, and 2161 patients received notifications of an irregular pulse).

General Data Protection Regulation (GDPR) requirements are fulfilled by the electronic informed consent form, which can also be adapted as needed for other potentially applicable data protection standards. Because health care professionals are not involved in PRO data collection, it would be beneficial for the results of the my ePRO app questionnaires to be transferred to the electronic health record of the patients. Current plans are for transfers of this sort to be enabled in Germany in 2021 through the provisions of the Digital Healthcare Act (DVG). Such a closed loop will ensure patient centricity and autonomy in data collection, storage, and sharing.

Some limitations of the DePRO study should be considered. First, the study only includes data from PROs. Second, the technology used to obtain data relies on the 2D matrix code of the outer drug package and not on a validated diagnosis by a health care professional. However, this study explicitly assesses the advantages and disadvantages of this way of capturing data. Third, it is possible that only technophile patients who are using the my ePRO app will decide to participate in the study. This may constitute a selection bias. Because T2DM affects predominantly older people and older people tend to be less inclined to use mobile devices and apps, the bias introduced may be considerable. In addition, it is unavoidable that even in the subgroup of technophile patients there might be relevant differences between those participating and those not participating. Fourth, among patients using the my ePRO app it will not be possible to distinguish for certain between patients using metformin and those not taking metformin, but documenting their health status within the my ePRO app. By using a recent drug package supplied by the pharmacist inviting the patient to the study, the medication intake behavior is an unverifiable variable. However, to prevent inappropriate participation, mitigation activities (checking 2D matrix codes for duplicate entries, blocking of the scanning tool after the start of documentation, and preventing patients from re-entering data after withdrawing consent) have been implemented in the my ePRO app. Fifth, this is a single-arm cohort study without a comparison group. However, the study is not designed to compare self-care behavior between my ePRO app users and other groups. By contrast, the study aims at understanding both the behavior and the outcomes of my ePRO app users, identified by the possession of metformin-containing drug packages, by investigating their my ePRO app data. Sixth, only patients using metformin will be able to participate in the study, precluding generalization of results to patients with other treatments. However, this study specifically aims to investigate self-care activities among patients with metformin-treated T2DM, a major subset of the overall T2DM population. Seventh, although the study aims to include participants from a variety of geographic regions, there may be local limitations that reduce the representativeness of patients recruited, such as patient access to recruiting pharmacists. Furthermore, recruitment through diabetes-focused pharmacies will limit the representativeness of the study population as a subset of the German diabetes population. Nevertheless the provision of the my ePRO app within public app stores may mean that the population sample is not limited to patients recruited in pharmacies—if the information that there is a study available spreads among patients, participants from a larger variety of geographic regions may be recruited. Finally, analysis results transferred to the sponsor will be anonymized; therefore, in cases of incomplete data there will be no possibility of contacting the patient for clarification and no possibility of performing source-data verification. Hence, it will be difficult to assess data quality.

### Conclusions

The design of the DePRO study represents a substantial advance in the evaluation of the digital capturing of PRO data.

## Appendix

Multimedia Appendix 1 My ePRO app screenshots.

Multimedia Appendix 2 Study questionnaire (German).

Multimedia Appendix 3 Study questionnaire (English).

## Abbreviations

DM: diabetes mellitus
DTSQ: Diabetes Treatment Satisfaction Questionnaire
EQ-5D-5L: 5-level, 5-dimension EuroQol Questionnaire
PRO: patient-reported outcome
QR: quick response
SDSCA: Summary of Diabetes Self-Care Activities
VAS: visual analog scale
