# Supplementary figures and images for: Evaluation of Self-Care Activities and Quality of Life in Patients With Type 2 Diabetes Mellitus Treated With Metformin Using the 2D Matrix Code of Outer Drug Packages as Patient Identifier: Protocol for the DePRO Proof-of-Concept Observational Study
Source: JMIR Res Protoc. 2021 Jan 11;10(1):e21727. doi: 10.2196/21727 (PMC7834935; doi:10.2196/21727)

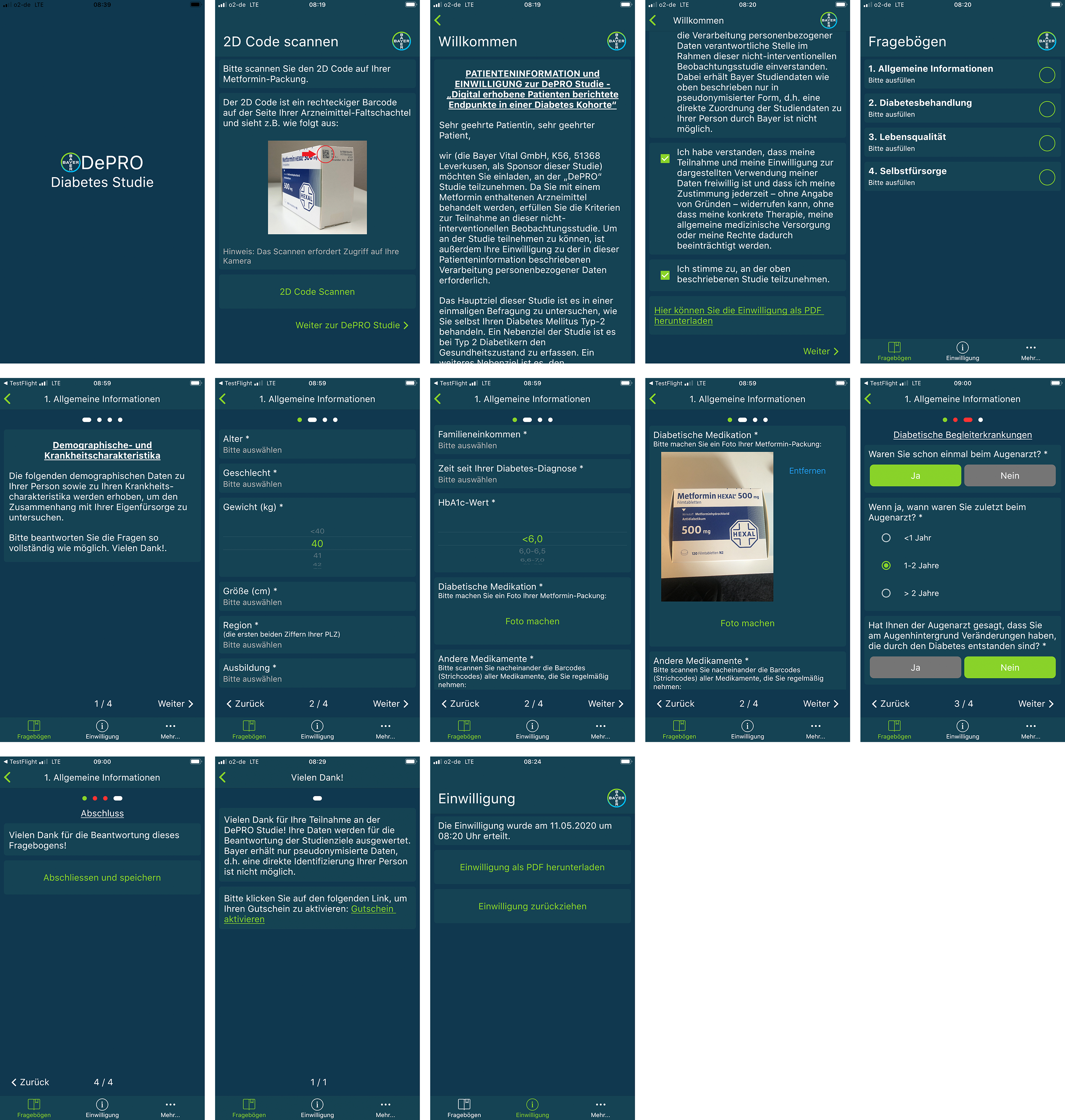

Supplement: Multimedia Appendix 1 [file resprot_v10i1e21727_app1.png]
